# Anakinra in cerebral haemorrhage to target secondary injury resulting from neuroinflammation (ACTION): Study protocol of a phase II randomised clinical trial

**DOI:** 10.1177/23969873231200686

**Published:** 2023-09-15

**Authors:** MP Cliteur, AG van der Kolk, G Hannink, J Hofmeijer, WMT Jolink, CJM Klijn, FHBM Schreuder

**Affiliations:** 1 Department of Neurology, Donders Institute for Brain, Cognition and Behaviour, Radboud University Medical Center, Nijmegen, The Netherlands; 2 Department of Medical Imaging, Radboud University Medical Center, Nijmegen, The Netherlands; 3 Department of Neurology, Rijnstate Hospital, Arnhem, The Netherlands; 4 Department of Clinical Neurophysiology, University of Twente, Enschede, The Netherlands; 5 Department of Neurology, Isala Hospital, Zwolle, The Netherlands

**Keywords:** Intracerebral haemorrhage, oedema, neuroinflammation, randomised trial, protocol

## Abstract

**Background:**

Inflammation plays a vital role in the development of secondary brain injury after spontaneous intracerebral haemorrhage (ICH). Interleukin-1 beta is an early pro-inflammatory cytokine and a potential therapeutic target.

**Aim:**

To determine the effect of treatment with recombinant human interleukin-1 receptor antagonist anakinra on perihematomal oedema (PHO) formation in patients with spontaneous ICH compared to standard medical management, and investigate whether this effect is dose-dependent.

**Methods:**

ACTION is a phase-II, prospective, randomised, three-armed (1:1:1) trial with open-label treatment and blinded end-point assessment (PROBE) at three hospitals in The Netherlands. We will include 75 patients with a supratentorial spontaneous ICH admitted within 8 h after symptom onset. Participants will receive anakinra in a high dose (loading dose 500 mg intravenously, followed by infusion with 2 mg/kg/h over 72 h; *n* = 25) or in a low dose (loading dose 100 mg subcutaneously, followed by 100 mg subcutaneous twice daily for 72 h; *n* = 25), plus standard care. The control group (*n* = 25) will receive standard medical management.

**Outcomes:**

Primary outcome is PHO, measured as oedema extension distance on MRI at day 7 ± 1. Secondary outcomes include the safety profile of anakinra, the effect of anakinra on serum inflammation markers, MRI measures of blood brain barrier integrity, and functional outcome at 90 ± 7 days.

**Discussion:**

The ACTION trial will provide insight into whether targeting interleukin-1 beta in the early time window after ICH onset could ameliorate secondary brain injury. This may contribute to the development of new treatment options to improve clinical outcome after ICH.

## Introduction

Spontaneous intracerebral haemorrhage (ICH) is a devastating subtype of stroke.^1^ Two distinct mechanisms contribute to brain injury in patients with ICH. First, extravasation of blood into the brain parenchyma results in direct damage by physical disruption of the brain’s cellular architecture and mass effect compressing surrounding tissue.^2–4^ Then, following this primary brain injury, an inflammatory response is triggered, consisting of microglia activation, blood brain barrier (BBB) degradation and influx of circulating inflammatory cells.^5^ This process is directed at resolving the haematoma and damaged tissue but simultaneously affects the adjacent viable brain parenchyma, resulting in secondary brain injury.^6^ Perihaematomal oedema (PHO) is considered an important imaging marker of secondary brain injury and is associated with clinical deterioration and worse functional outcome.^7–10^ PHO starts to develop during the first 24 h after ICH onset, and continues to increase during the first 7–10 days.^11,12^

The pro-inflammatory cytokine interleukin-1 beta (IL-1β) has been identified as an attractive candidate to target secondary brain injury after ICH. In the brain, IL-1β originates from macrophages and it is activated by microglia and astrocytes.^13^ It has several biological effects that include upregulation of pro-inflammatory cytokines, stimulation of matrix metalloproteinases production and leucocyte infiltration and activation of microglia.^13,14^ In a systematic review and meta-analysis of studies reporting molecular markers of inflammation in human brain tissue, we found that levels of IL-1β were consistently increased within 6–12 h after ICH onset.^15^ Moreover, elevated IL-1β levels in animal serum and human cerebrospinal fluid have been found to be associated with increased PHO formation.^16,17^

IL-1β is antagonised by the naturally occurring interleukin-1 receptor antagonist (IL-1ra),^18^ and higher levels of autologous IL-1ra after ICH have been associated with a reduction in PHO.^19^ Recombinant human IL-1ra (anakinra) has been available for treatment of inflammatory disease such as rheumatoid arthritis since 2001. Preclinical data support a neuroprotective therapeutic potential of anakinra in both ischaemic and haemorrhagic stroke. In a meta-analysis of preclinical stroke studies, IL-1ra treatment reduced infarct volume by 36% in rodents.^20^ To date, two clinical studies have investigated the use of anakinra in patients with acute stroke.^21,22^ Both trials indicated a significant reduction of serum inflammatory markers without safety concerns.^21,22^ Amongst the 119 participants, only five patients with ICH were included in these two trials.^21^ Recently, a first randomised trial testing anakinra in 25 patients with spontaneous ICH was published (BLOC-ICH).^23^ They could demonstrate feasibility of anakinra treatment within 8 h after ICH onset but were unable to determine the effect on PHO formation due to limited sample size. Hence, the effect of anakinra on perihematomal inflammation after spontaneous ICH and the development of secondary brain injury remains unclear.

The Anakinra in Cerebral haemorrhage to Target secondary Injury resulting frOm Neuroinflammation (ACTION) trial aims to establish whether administration of anakinra in patients with ICH affects PHO formation. We hypothesise that anakinra safely reduces PHO after ICH, and that its effect is dose-dependent.

## Methods

### Trial design

ACTION (www.action-study.nl) is an investigator-initiated, phase II, prospective, randomised trial with open label treatment and blinded end-point assessment (PROBE). There will be three arms (1:1:1). In the first arm, participants will receive a high-dose of intravenous anakinra in addition to standard medical management, in the second arm, a subcutaneous low-dose of anakinra in addition to standard medical management, and the third arm constitutes of standard medical management without anakinra. Patients in all arms will get standard medical management. Each treatment arm will include 25 patients. The primary aim of the trial is to study whether use of anakinra has an effect of PHO compared to standard medical management; for this analysis we will combine the two anakinra treatment arms. In the secondary analysis we will investigate whether a difference in dosage of anakinra modifies the effect of anakinra on PHO. Main reasons for studying whether such a difference exists, is that the subcutaneous administration of anakinra is easier in use and has lower costs than the intravenous administration, which on the other hand might be more effective.

The study will be conducted according to the principles of the Declaration of Helsinki and in accordance with the Medical Research Involving Human Subjects Act (WMO). The study was approved by the medical ethics committee of the region Arnhem-Nijmegen (reference NL76607.091.21).

### Participants

Participants will be recruited from the emergency wards of three Dutch hospitals (Radboud University Medical Center Nijmegen, Rijnstate Hospital Arnhem and Isala Hospital Zwolle) over the course of a 2-year period. Members of the study team are listed in Appendix 1. To be eligible to participate in this study participants must meet all of the inclusion criteria and none of the exclusion criteria, listed in Table 1.

**Table 1.** Inclusion and exclusion criteria.

Inclusion criteria	Exclusion criteria
1. Age ⩾18 years; 2. Supratentorial non-traumatic ICH confirmed by CT, without a confirmed causative lesion on admission CTA (e.g. aneurysm, AVM, DAVF, cerebral venous sinus thrombosis) or other known underlying lesion (e.g. tumour, cavernoma); 3. Minimal ICH volume of 10 mL; 4. Intervention can be started within 8 h of symptom onset; 5. Patient’s or legal representative’s informed consent.	1. Severe ICH, unlikely to survive the first 72 h (defined as GCS score <6 at time of consent); 2. Confirmed or suspected haemorrhagic transformation of an arterial or venous infarct; 3. Planned neurosurgical haematoma evacuation; 4. Severe infection at admission, requiring antibiotic treatment; 5. Known active tuberculosis or active hepatitis; 6 Use of immunosuppressive or immune-modulating therapy at admission; 7. Neutropenia (defined as an ANC <1.5 × 10^9^/L); 8. Pre-stroke mRS score ⩾3; 9. Pregnancy or breast-feeding; 10. Standard contraindications to MRI; 11. Known prior allergic reaction to gadolinium contrast or one of the constituents of its solution for administration; 12. Known allergy to anakinra or other products that are produced by DNA technology using the micro-organism *E. coli*; 13. Vaccinations with live attenuated micro-organisms within the last 10 days prior to the ICH; 14. Severe renal impairment (eGFR <30 mL/min/1.73 m^2^); 15. Known active malignancy.

ANC: absolute neutrophil count; AVM: arteriovenous malformation; CT: computed tomography scan; CTA: computed tomography angiography scan; DAVF: dural arteriovenous fistula; eGFR: estimated glomerular filtration rate; GCS: Glasgow Coma Scale; ICH: intracerebral haemorrhage, MRI: magnetic resonance imaging; mRS: modified Rankin Scale.

### Randomisation

Participants will be randomised in the ratio of 1:1:1 via a secure web-based internet system (CastorEDC). Treatment allocation will be stratified for centre of participation, admission ICH volume (10–30 mL vs >30 mL as estimated with the ABC/2 method on non-contrast CT at admission) and renal function (estimated glomerular filtration rate (eGFR) 30–49 vs >50 mL/min/1.73 m^2^) to ensure balance in factors associated with development of PHO and anakinra plasma concentrations. Treatment allocation is not blinded to participants nor to study team members, compatible with the open-label treatment in the PROBE design. Instead, outcome assessment will be blinded for treatment allocation.

### Intervention

Patients will be randomised between three study arms:

1. High-dose anakinra group: within 8 h of symptom onset, patients will receive an intravenous anakinra loading dose of 500 mg. The loading dose will be directly followed by continuous intravenous infusion of anakinra at 2 mg/kg/h for 72 h. All patients will also receive standard care according to the Dutch stroke guideline.
2. Low-dose anakinra group: within 8 h of symptom onset patients will receive a subcutaneous anakinra loading dose of 100 mg, followed by subcutaneous injection of 100 mg twice daily for 72 h (i.e. patients will receive a total of six injections). The twice daily subcutaneous administration of anakinra will be timed at 07:00 and 19:00 h (±2 h) with a minimum of 6 and a maximum of 18 h between loading dose and the second dose. All patients will also receive standard care.
3. Standard care group: patients will be admitted to the specialised stroke unit or medium/intensive care unit depending on their clinical condition. They will be treated in accordance to the Dutch stroke guidelines, which includes intensive neurological monitoring, blood pressure management, prevention of complications such as aspiration and infections, and specialised stroke rehabilitation.

The high-dose anakinra regimen is based on the dosage in two previous described phase II trials, which both used an intravenous bolus injection of 100 mg followed by continuous infusion of 2 mg/kg/h for a period up to 72 h.^21,24^ This dosage was found to be safe and penetrated the BBB to achieve therapeutic concentration within 3 h of commencement of infusion in experimental studies in subarachnoid haemorrhage patients. For this study, we chose a higher loading dose of 500 mg based on pharmacokinetic data. A loading dose of 500 mg results in an effective intrathecal concentration within 45 min, whereas 100 mg is expected to reach an effective intrathecal concentration in 104 min.^25^ The percentage transfer of anakinra across the BBB under physiological conditions is approximately 1%.^26^ However, after ICH the BBB integrity in the perihematomal region is likely to be compromised and higher concentrations of anakinra might enter the brain parenchyma. This rationale supports the use of a lower anakinra dose. The low dose regime in this study will consist of a subcutaneous anakinra bolus injection of 100 mg, followed by subcutaneous injection of 100 mg twice a day for 3 days. This dose was well tolerated without any safety concerns in a previously phase-II trial.^22^

### Study parameters

**Figure 1.**
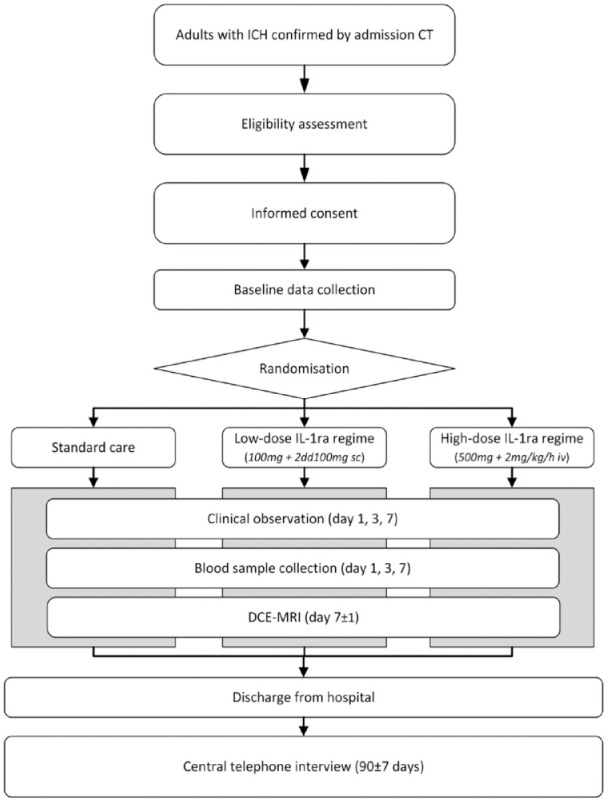
Study procedures. CT: computed tomography scan; ICH: intracerebral haemorrhage; IL-1ra: interleukin-1 receptor antagonist; DCE-MRI: dynamic contrast enhanced magnetic resonance imaging.

In addition to the administration of study treatment, patients will undergo study procedures at the day of admission (day 0), day 1 (±12 h), day 3 (±12 h), day 7 (±1 day) and day 90 (±7 days) after ICH onset. The study procedures are schematically visualised in Figure 1 and Table 2. Variables that will be obtained are:

**Table 2.** Schedule of study assessments.

	Admission	Screening and consent	Day 0	Day 1	Day 2	Day 3	Day 7	Day 90 ± 7
Admission brain CT/CTA	X							
Renal function	X							
NIHSS and GCS	X			X		X	X	
Medical history		X						
Confirmation of eligibility		X						
Informed consent		X						
Randomisation		X						
Blood sampling			X	X		X	X	
Loading dose anakinra			X					
Anakinra administration			X	X	X	X		
AESI/SAE check				X	X	X	X	X
Brain DCE-MRI							X	
Clinical outcome assessment								X

AESI: adverse event of special interest; CT: computed tomography scan; CTA: computed tomography angiography scan; DCE-MRI: dynamic contrast enhanced magnetic resonance imaging; GCS: Glasgow Come Scale; NIHSS: National Institutes of Health Stroke Scale; SAE: serious adverse event.

#### Clinical data

We will collect data on the index ICH (including date and time of onset, date and time of presentation at the emergency ward, location and volume of the ICH assessed on baseline CT), baseline physical examination (including admission blood pressure, Glasgow Coma Scale (GCS) and National Institutes of Health Stroke Scale (NIHSS) scores), previous medical history (including cardiovascular risk factors, medication use and premorbid modified Rankin Score (mRS) score), body weight and height. During hospital stay, we will record NIHSS and GCS at day 1, day 3 and day 7. Details of the study drug administration (including date and time), and the use of concomitant medication during hospital stay will also be recorded.

#### Blood sampling

We will collect baseline blood levels of C-reactive protein, leucocyte count, serum glucose levels, creatinine, and eGFR. Serum and whole blood samples will be obtained at baseline, day 1, 3 and 7 to determine the level of IL-1β, IL-6, high-sensitivity C-reactive protein, neutrophil and total white blood cell counts. The whole blood samples will be used for metabolomic profiling.

#### MRI data acquisition

At day 7 ± 1 participants will undergo one 3 Tesla (3 T) MRI examination of the brain. The MRI includes structural imaging (T_1_-weighted, fluid-attenuated inversion recovery (FLAIR), susceptibility-weighted imaging (SWI)) and dynamic contrast-enhanced (DCE) imaging to assess leakage of the BBB as a consequence of neuroinflammation. This DCE-MRI sequence consists of a baseline dynamic T_1_-weighted imaging before contrast administration of the entire brain, short T_1_-weighted imaging of the superior sagittal sinus during contrast injection to obtain a venous input function and 12 repetitions of the dynamic T_1_-weighted imaging after the initial contrast agent passage of the entire brain again. Acquisition parameters are summarised in Table 3.

**Table 3.** MRI sequence parameters.

Sequence	T_1_ MPRAGE	T_2_ FLAIR	SWI	T_1_ mapping	DCE-MRI pre	DCE-MRI bolus	DCE BBB leakage
Acquisition type	3D GRE	3D TSE	3D GRE	3D GRE	3D SR GRE	3D SR GRE	3D SR GRE
Field of view (mm)	240 × 240 × 173	250 × 250 × 176	250 × 250 × 156	230 × 230 × 176	256 × 256 × 144	256 × 256 × 30	256 × 256 × 144
Acquired spatial resolution (mm)	0.9 × 0.9 × 0.9	1.0 × 1.0 × 1.0	1.0 × 1.0 × 3.0	1.4 × 1.4 × 2.0	1.0 × 1.0 × 2.0	1.0 × 1.0 × 3.0	1.0 × 1.0 × 2.0
Orientation	Sagittal	Sagittal	Transverse	Sagittal	Sagittal	Sagittal	Sagittal
TR (ms)	2300	5000	27	15	296	344	296
TE (ms)	2.32	394	20	4.14	1.56	1.56	1.56
TI (ms)	900	1800	N/A	N/A	120	120	120
Flip angle (°)	8	N/A	15	2, 5, 10 & 20	20	20	20
No. of dynamic acquisitions	N/A	N/A	N/A	N/A	5	38	12
NSA	1	1	1	1	1	1	1
Scan time (min:s)	5:21	3:42	2:43	1:35	2:10	1:58	5:13

MPRAGE: magnetisation prepared rapid gradient echo; GRE: gradient recalled echo; FLAIR: fluid-attenuated inversion recovery; MPRAGE: magnetisation prepared rapid gradient echo imaging; MRI: magnetic resonance imaging; N/A: not applicable; NSA: number of signal averages; SWI: susceptibility weighted imaging; TE: echo time; TI: inversion time; TR: repetition time.

#### MRI data analysis

**Figure 2.**
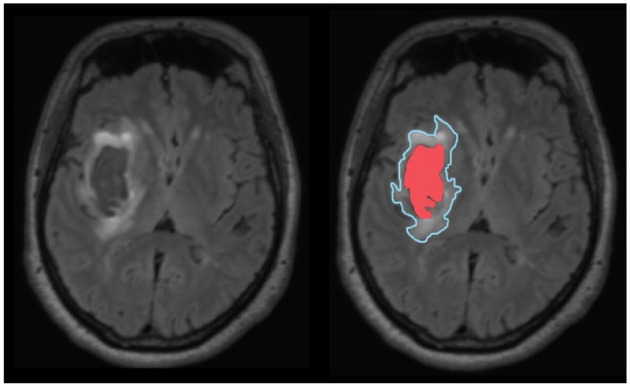
Example of the delineation of perihaematomal oedema (blue line) and the intracerebral haematoma (depicted in red) on a MRI T2-FLAIR scan.

Image assessment will be performed by trained readers blinded to treatment allocation and baseline characteristics. All acquired sequences will be co-registered to the T_1_-weighted MPRAGE sequence to enable optimal comparisons. First, the haematoma will be manually annotated on all available slices using the T_1_-weighted and SWI images. Haemorrhage is defined as areas with hyperintense signal on T_1_-weighted images with corresponding hypointense signal on SWI images. PHO will be assessed using the FLAIR images, with PHO defined as the T_2_ hyperintensity surrounding the haematoma. Both areas will be delineated using computer-assisted image segmentation (see Figure 2), enabling calculation of the volume of PHO and haematoma. For analysis, the oedema extension distance (OED) in cm will be calculated as the difference in radius of a sphere with the volume of PHO + haematoma and the radius of a sphere with the volume of haematoma alone $\left(OED=\sqrt[{3}]{\frac{PHO\;volume+ICH\;volume}{\frac{4}{3}\pi }}-\sqrt[{3}]{\frac{ICH\;volume}{\frac{4}{3}\pi }}\right).$ ^27^ To assess BBB integrity, we will calculate the BBB transfer constant K^trans^ using a two-compartment general kinetic model to represent tracer dynamics measured in the vascular space and extracellular space.^28^

#### Functional outcome

Functional outcome will be assessed at day 90 (±7 days) via telephone interview by trained research personnel blinded to treatment allocation. Standardised reports using the mRS, Barthel Index (BI) and the EQ-5D-5L quality of life questionnaire will be used.

### Outcomes

The primary outcome measure will be PHO measured as the OED^27^ in cm on MRI at day 7 ± 1.

Secondary outcomes are:

1. The safety profile of anakinra in patients with ICH.
2. BBB disruption on DCE-MRI on day 7 ± 1;
3. Change in levels of serum inflammatory markers from baseline to day 1, 3 and 7; and
4. Functional outcome at 90 days.

### Adverse event reporting

Adverse events (AEs) are defined as any undesirable experience occurring to a subject during the study, whether or not considered related to the administration of anakinra or other study procedures. Serious adverse events (SAEs) are AEs that result in death, are life-threatening, require hospitalisation or prolongation of existing hospitalisation, result in persistent or significant disability, are congenital anomalies or birth defects, or other important medical events, based on the judgement by the study team. All (S)AEs reported spontaneously by the subject or observed by the investigator will be recorded. Despite the excellent safety record of anakinra and the relatively high number of complications of the disease under study, we will identify the following ‘adverse events of special interest’ (AESIs) to assess participants’ tolerability to anakinra:

1. *Early neurological deterioration*, defined as *a* ⩾ 2 point decrease in GCS or a ⩾4 point increase in the NIHSS score sustained for at least 8 h, within the first 3 days of hospitalisation;
2. *Infection*, categorised as urinary tract infection, pneumonia, bloodstream infection, or other infections. Infections will be diagnosed and categorised based on the Centers of Disease Control and Prevention (CDC) definitions^29^;
3. *Skin injection reaction*, defined as one or more of the following: erythema, ecchymosis, inflammation, and severe pain;
4. *Allergic reaction*, defined as an untoward reaction to anakinra developing over minutes to days after treatment initiation, and is clinically characterised by skin-mucosal, respiratory and/or cardiovascular symptoms. This does not include known side effects of anakinra;
5. *Anaphylaxis*, which is considered when two or more of the following occur during an allergic reaction: generalised involvement of the skin-mucosal tissue, respiratory compromise, reduced blood pressure or associated symptoms, or persistent gastrointestinal symptoms;
6. *Neutropenia*, defined as an absolute neutrophil count (ANC) <1.5 × 10^9^/L.

All SAEs and AESI reported spontaneously by the subject or observed by the investigator will be recorded from baseline to day 90. Specific urgent medical reasons to withdraw from the trial have been defined and will be monitored closely: neutropenia, neurosurgical haematoma evacuation, anaphylaxis after anakinra administration, and severe clinical deterioration in which the condition of the participant is considered to be moribund and active treatment is discontinued.

### Outcome adjudication

An outcome adjudication committee will adjudicate SAEs and AESI on a regular interval. This committee will consist of two experienced vascular neurologists. The committee will receive outcome information blinded to treatment allocation.

### Data and safety monitory board

Study safety will be monitored by an independent data and safety monitory board (DSMB) consisting of a neurosurgeon, a neurologist, and a biostatistician. Interim analyses will be performed after completion of the follow-up period of 24 and 48 participants and will assess the adjudicated events to evaluate safety of the intervention, compared to standard care. The stopping rule for premature ending of the trial because of safety reasons is at least two times (i.e. ⩾200%) higher proportion of participants with an urgent medical reason for withdrawal in the two intervention arms combined and compared to the comparator arm (2:1), with at least two events per group. Results of the interim analyses can be compared between the high dose and low dose treatment arms, to advise on termination of a single treatment arm in case ⩾75% of the withdrawals should occur in one treatment arm.

### Statistical methods

The primary analysis of this trial will be conducted under the principle of intention-to-treat. Baseline characteristics of the patients included in each treatment group will be reported and tabulated to demonstrate group comparability. For the primary analysis, we will use a two-sided independent t-test to determine a mean difference and 95% CI between the OED at MRI on day 7 ± 1 of the two anakinra treatment groups combined and the standard care group. In an exploratory analysis, we will determine whether a difference in OED exists between the high dose and low dose anakinra group, again using a two-sided independent *t*-test.

Safety analyses will include all SAEs and AESIs up to 90 days after randomisation. To determine the safety profile of anakinra between the three treatment arms, we will compare the number of AESIs and urgent medical reasons for study withdrawal between the three treatment arms in 2 × 3 tables, tested by the Fisher exact probability test. To assess BBB disruption, difference in the K^trans^ between the three treatment groups will be analysed with ANOVA.

To determine the effect of anakinra on the time-dependent profile of serum inflammatory markers, we will use a linear mixed model to assess the effect of anakinra dosage on the area under the curve for these metabolites compared to standard care. The effect of the treatment on the distribution of the mRS at 90 ± 7 days will be analysed using ordinal logistic regression yielding common odds ratio’s with corresponding 95% CI. We will use a two-sided independent t-test to determine a mean difference and 95% CI between the Barthel Index of the two anakinra treatment groups combined versus the standard medical management group.

### Sample size calculation

The OED is known to be independent of haematoma size, with a normal distribution. Based on the available preclinical and clinical data we assume an OED of 0.60 (SD 0.25) at day seven in the standard care group and estimate a reduction of OED from 0.60 to 0.40 (SD 0.25) in the anakinra treated group (high and low dose groups combined).^30^ With a group size ratio of 1:2 for control versus treatment, a power of 0.80, and an alpha of 0.05, a sample size of 19 patients in the control group, and of 39 in the intervention group is required. Assuming a 25% drop-out rate due to the severity of ICH, the final sample size is 25 patients for the control group and 50 patients for the intervention group, resulting in a total sample size of 75 patients for this trial.

### Trial status

Patient recruitment started in July 2022 and is expected to finish in the last quarter of 2024.

## Discussion

ACTION is a multicentre randomised phase II trial studying the effect of anakinra on PHO development in patients with spontaneous supratentorial ICH. The primary aim of this trial is to establish whether administration of anakinra can reduce PHO as measured by the OED. In addition, we will assess the safety profile of anakinra in patients with ICH, and whether use of anakinra affects serum inflammation markers, BBB integrity, and clinical outcome during follow-up.

Experimental and clinical evidence indicates that targeting IL-1β is a promising therapeutic strategy in stroke patients. For this purpose, anakinra is an attractive option, as it is a highly selective IL-1ra that penetrates into the central nervous system and has an excellent safety profile. However, the optimal dosage of anakinra in patients with ICH is still unclear. The two clinical trials that have investigated the use of anakinra in patients with acute stroke have studied different dose regimes. The first trial used a high-dose regime consisting of a 100 mg loading dose followed by continuous infusion of 2 mg/kg/h for 3 days,^21^ whereas the second trial used a low-dose anakinra regime of 100 mg twice daily for 3 days.^22^ Anakinra was found to be safe and well tolerated in both trials and markedly attenuated the levels of circulating inflammatory molecules such as CRP and IL-6 levels.

Under physiological conditions approximately 1% of anakinra crosses the BBB,^25^ which would imply that administration of a high dosage of anakinra is probably needed for clinically relevant levels in the brain. However, after ICH, higher concentrations of anakinra are expected to enter the brain parenchyma adjacent to the haematoma since the BBB integrity in the perihaematomal region is compromised. This might allow a lower dose regime with improved patient tolerance at significant lower cost. The three-armed design of ACTION may help to design future phase III trials by estimation of effect sizes and comparison of the high intravenous and low subcutaneous anakinra dosage. The primary analysis will provide evidence as to whether anakinra can influence the development of secondary brain injury, while secondary analysis will assist determining the required dose regimen. In addition, we use advanced DCE-MRI to quantify BBB disruption in the perihaematomal region as an indirect marker of neuroinflammation.

A potential limitation of our study is the relatively limited sample size. The high mortality rate after ICH might lead to a substantial drop-out of study participant that are not able to undergo a MRI examination at 7 ± 1 days. Although our sample size would allow for a 25% drop-out, this might be higher resulting in decreased statistical power. Moreover, the estimated effect size is based on preclinical data which might not translate to a human study. Using OED as our metric for PHO in our primary outcome analysis aims to mitigate the required sample size, as it is not affected by haematoma volume and thus strongly reduces the required sample size in comparison with using absolute or relative PHO.^27^

One other randomised phase II study has investigated anakinra to reduce PHO in patients with ICH (BLOC-ICH).^23^ In this placebo-controlled trial, patients with spontaneous supratentorial ICH were randomised to receive either 100 mg of anakinra subcutaneously twice daily during the 72 h after ICH onset, or placebo. Primary outcome was OED on CT imaging obtained at day 3 after symptom onset. Secondary outcomes included serum inflammation markers and functional outcome at 3 months. Patient recruitment was severely impacted by the COVID-19 pandemic and the trial had to be ceased prematurely after inclusion of 25 of the intended 80 participants. Although this trial provides preliminary data on the safety and feasibility of anakinra in acute ICH patients, it lacked statistical power to determine the effect of anakinra on perihaematomal oedema formation. We will collaborate with the investigators of BLOC-ICH to combine data into an individual patient data meta-analysis of patients treated with anakinra after ICH once the ACTION trial has been completed.

In conclusion, the ACTION trial has the potential to offer valuable insights into the role of neuroinflammation in the development of secondary brain injury after ICH, and whether this can be therapeutically modulated. Moreover, it will help determine the feasibility and design of future phase III trials of immunomodulatory therapies in patients with ICH.

## Appendix 1

### Local investigators:

- Isala Hospital, Zwolle: W.M.T. Jolink, R. Eilander, E. Ponjee, H.Z. Flach
- Radboud University Medical Center, Nijmegen: M.P. Cliteur, R. Hoogland-Franssen, C.J.M. Klijn, F.H.B.M. Schreuder
- Rijnstate Hospital, Arnhem: J. Hofmeijer, M.J. van Gorp, I. Visser

### Executive committee:

M.P. Cliteur, C.J.M. Klijn, F.H.B.M. Schreuder (Radboud University Medical Center)

### Outcome adjudication committee:

J.M. Coutinho (Amsterdam University Medical Center); H.B. van der Worp (UMC Utrecht)

### Data Safety and Monitoring Board

W.C. Peul (Leiden University Medical Center; Chair), A.R. Parry-Jones (Salford Royal NHS); J. in ‘t Hout (Radboud University Medical Center)
